# Commentary: Transcription factor MEF2D regulates aberrant expression of ACSL3 and enhances sorafenib resistance by inhibiting ferroptosis in HCC

**DOI:** 10.3389/fphar.2025.1575998

**Published:** 2025-10-13

**Authors:** Xiaoyan Chen, Jin Xie, Wei Fan, Junfeng Zhang, Feng Gao, Zhi Li

**Affiliations:** ^1^ School of Basic Medical Sciences, Hubei University of Chinese Medicine, Wuhan, China; ^2^ Hubei University of Chinese Medicine, Wuhan, China; ^3^ Taihe Hospital, Hubei University of Medicine, Shiyan, China; ^4^ First School of Clinical Medicine, Hubei University of Medicine, Shiyan, China; ^5^ Interventional Cancer Institute of Chinese Integrative Medicine, Putuo Hospital, Shanghai University of Traditional Chinese Medicine, Shanghai, China

**Keywords:** hepatocellular carcinoma, sorafenib resistance, Mef2d, ACSL3, ferroptosis

## Introduction

**FIGURE 1:**
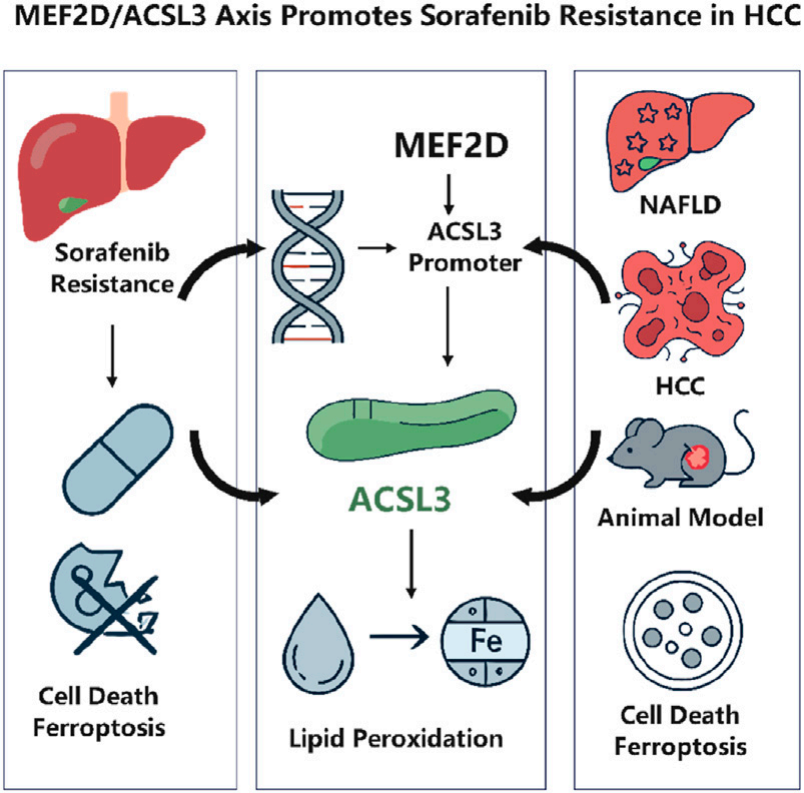
MEF2D drives sorafenib resistance in hepatocellular carcinoma by transcriptionally upregulating ACSL3 to suppress ferroptosis. *Created with* BioRender.com.

Hepatocellular carcinoma (HCC) is a highly fatal cancer globally. Sorafenib, as a primary treatment agent, encounters substantial constraints owing to drug resistance, which significantly undermines its clinical effectiveness (Sun et al., 2022). A new study in Frontiers in Pharmacology has demonstrated for the first time that the transcription factor MEF2D inhibits ferroptosis by upregulating long-chain ACSL3, therefore facilitating sorafenib resistance in HCC. This study combines clinical data, animal models, and cellular investigations to validate the oncogenic function of ACSL3 in the transition from NAFLD to HCC. It clarifies the molecular mechanism via which MEF2D directly interacts with the ACSL3 promoter to modulate its transcription (Li et al., 2024). This article offers a comprehensive analysis of the study, addressing its scientific importance and possible translational implications (Figure 1).

## Background

**FIGURE 2:**
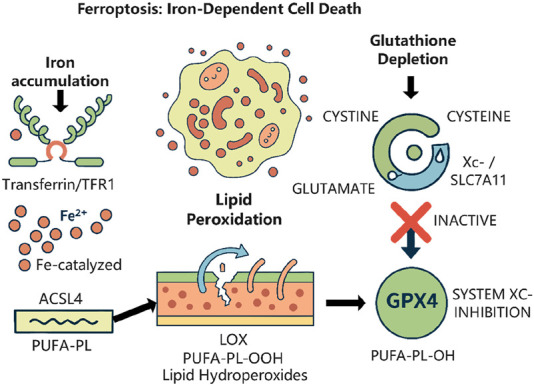
The core regulatory mechanism of ferroptosis. This form of regulated cell death is driven by the iron-dependent accumulation of lipid peroxides. *Created with* BioRender.com.

The progression of HCC is intricately associated with chronic liver conditions, including viral hepatitis, alcoholic liver disease, and NAFLD (Hyun et al., 2021). Sorafenib, a multi-targeted tyrosine kinase inhibitor, prolongs patient longevity by obstructing angiogenesis and tumour growth. Nevertheless, extensive drug resistance results in unfavourable outcomes (Li et al., 2023). Recent findings indicate that sorafenib can trigger ferroptosis (Figure 2), a type of cell death driven by iron-dependent lipid peroxidation, and that drug resistance may be linked to the suppression of this process (Hao et al., 2024; Xu et al., 2023). The principal characteristic of ferroptosis is the abnormal buildup of lipid reactive oxygen species (ROS) and the deactivation of antioxidant mechanisms such as GPX4 (Chen et al., 2021). Lipid metabolism reprogramming is essential in modulating ferroptosis, as the ACSL family affects cell membrane phospholipid composition through the catalysis of fatty acid activation (Cai et al., 2023). ACSL3 has been documented to impede ferroptosis by promoting monounsaturated fatty acids and diminishing polyunsaturated fatty acid peroxidation (Cao et al., 2025); however, its precise function in HCC is yet to be elucidated. MEF2D, a member of the myocyte enhancer factor family, is aberrantly overexpressed in multiple malignancies, facilitating tumour proliferation, invasion, and drug resistance through the regulation of downstream genes (Surace et al., 2024). The role of MEF2D in regulating ferroptosis in HCC is yet uncertain.

### Key evidence and findings

This work examined the GSEA database and clinical samples, demonstrating that ACSL3 is markedly overexpressed in NAFLD and HCC tissues, with a positive correlation to disease severity (Li et al., 2024). Single-cell sequencing revealed that ACSL3 is primarily concentrated in hepatocytes, implying its potential role in facilitating the transition from NAFLD to HCC by modulating hepatic lipid metabolism. In animal studies, mice with high-fat diet-induced NAFLD demonstrated increased liver ACSL3 expression, alongside lipid vacuolization and intensified inflammation, hence reinforcing its pro-oncogenic function (Mossmann et al., 2023). Utilising siRNA knockdown and overexpression methodologies, researchers determined that ACSL3 mitigates sorafenib-induced lipid peroxidation and ROS generation by downregulating the ferroptosis-promoting regulator ACSL4 and upregulating the anti-ferroptotic proteins GPX4 and FTH1(12). This corresponds with ACSL3’s established role in catalysing MUFA to produce protective membrane phospholipids and, for the first time, distinctly delineates ACSL3’s inhibitory effect on ferroptosis in HCC. ChIP and luciferase reporter tests validated that MEF2D directly interacts with the MEF2 response element (MRE) in the ACSL3 promoter region, hence enhancing its transcription (Li et al., 2024). Clinical data analysis revealed a substantial positive connection between MEF2D and ACSL3 expression in HCC, with elevated levels of both correlating with unfavourable patient outcomes (Li et al., 2024). Furthermore, the suppression of MEF2D negated the ferroptosis inhibition and sorafenib resistance induced by ACSL3, underscoring the pivotal function of the MEF2D-ACSL3 axis. This study establishes a connection between the MEF2D-ACSL3 axis and the prevention of ferroptosis, as well as sorafenib resistance, for the first time, addressing a gap in the mechanisms of resistance in HCC. Utilising multi-omics analysis and experimental validation, this study elucidates ACSL3’s dual function in the evolution of NAFLD-HCC and suggests targeting MEF2D or ACSL3 as a novel approach to counteract sorafenib resistance, offering potential translational significance (Table 1).

**TABLE 1:** Summary of recent findings on the role of ACSL3 in NAFLD and HCC.

Key finding	Disease context	Mechanism, evidence, and supporting literature
ACSL3 is overexpressed in NAFL/NAFLD and HCC tissues and correlates with disease severity	NAFLD and HCC	Analysis of clinical tissue samples and GSEA databases demonstrates high ACSL3 expression (Li et al., 2024; Cai et al., 2023). Single-cell sequencing has identified hepatocytes as the primary cellular source of ACSL3 (Li et al., 2024)
ACSL3 promotes the transition from NAFLD to HCC by driving lipid accumulation	NAFLD-HCC Transition	In high-fat diet-induced NAFLD mouse models, increased liver ACSL3 expression is concurrent with lipid vacuolization and inflammation (Mossmann et al., 2023). *In vitro*, silencing ACSL3 in hepatocytes reduces oleic acid-induced lipid accumulation and steatosis, implicating it in driving steatosis (Klasson et al., 2022)
ACSL3 confers sorafenib resistance by inhibiting ferroptosis	HCC (Drug Resistance)	ACSL3 knockdown enhances cellular sensitivity to sorafenib (Li et al., 2024). Mechanistically, ACSL3 inhibits ferroptosis by downregulating the pro-ferroptotic regulator ACSL4 while upregulating anti-ferroptotic proteins GPX4 and FTH1, thereby reducing lipid peroxidation and reactive oxygen species (Li et al., 2024; Hao et al., 2024; Xu et al., 2023)
The MEF2D-ACSL3 axis is a key regulatory pathway in HCC.	HCC (Regulation)	The transcription factor MEF2D directly binds to the ACSL3 promoter region to enhance its transcription, as confirmed by ChIP and luciferase reporter assays (Li et al., 2024). Clinical data reveal a significant positive correlation between MEF2D and ACSL3 expression in HCC, with high levels of both predicting poor patient outcomes (Li et al., 2024)
ACSL3 modulates lipid metabolism to promote HCC progression	HCC (Metabolism)	ACSL3 promotes HCC cell proliferation by reprogramming fatty acid metabolism, particularly by catalyzing the production of monounsaturated fatty acids (MUFAs) used for membrane synthesis and energy storage (Li et al., 2023; Cai et al., 2023). It also contributes to HCC progression by modulating cholesterol homeostasis, which affects the tumor microenvironment and signaling pathways (Huang et al., 2023)

## Limitations and future directions

While this study provides significant insights into the MEF2D-ACSL3 axis, its limitations also illuminate critical and exciting avenues for future research. Rather than being constraints, these points represent a strategic roadmap for the scientific community to build upon our findings and develop robust therapeutic strategies against sorafenib resistance. Specifically, the reliance on a single-center cohort tempers the immediate clinical applicability of our findings, as the prevalence and importance of this axis may differ across more diverse patient populations, a critical factor for designing successful, broad-based clinical trials. Similarly, the absence of definitive genetic validation from knockout models means our therapeutic hypothesis requires more rigorous preclinical confirmation to de-risk the substantial investment required for human studies. These translational gaps underscore that moving from a compelling biological mechanism to a viable therapeutic intervention requires a dedicated phase of validation to ensure both safety and efficacy. For instance, our initial findings, based on a single-center cohort with limited NAFLD-related HCC cases, present an important opportunity for future multi-center studies to validate the generalizability of our results and dissect how the MEF2D-ACSL3 axis varies across different HCC etiologies. Similarly, advancing our *in vivo* work with more sophisticated models, such as hepatocyte-specific conditional knockouts for *Mef2d* or *Acsl3* in NAFLD-HCC mice, is essential for definitively dissecting the axis’s role in a context that more accurately simulates human disease progression. Furthermore, our work invites deeper mechanistic investigations by experts in epigenetics to explore the roles of DNA methylation and histone acetylation in MEF2D’s regulation of ACSL3, as well as the precise molecular cascade by which ACSL3 modulates its downstream targets ACSL4 and GPX4. Looking ahead, a comprehensive strategy is needed to map the upstream signaling pathways regulating MEF2D, such as Hippo-YAP or MAPK, and to identify other downstream effectors of ACSL3. This presents a clear call to action for medicinal chemists and pharmacologists to develop specific MEF2D or ACSL3 inhibitors and assess their efficacy in combination with sorafenib. Moreover, critical questions remain regarding the interplay between ferroptosis and other cell death pathways like autophagy and apoptosis, the influence of lipid availability in the tumor microenvironment on ACSL3 activity, and the dynamic evolution of the MEF2D-ACSL3 axis during long-term treatment leading to acquired resistance. Collectively, these directions provide a comprehensive framework for collaborative efforts across disciplines, aimed at systematically dismantling this resistance pathway and establishing new, more effective therapeutic paradigms for HCC.

## Discussion

This research elucidates the critical function of the MEF2D-ACSL3 axis in sorafenib resistance in hepatocellular carcinoma, providing novel insights into the relationship between tumour metabolism and cellular apoptosis. ACSL3, a pivotal enzyme in lipid metabolism, exhibits a dual function—facilitating cancer development while suppressing ferroptosis—highlighting the intricacies of metabolic reprogramming in tumour advancement and medication resistance. Extending the function of MEF2D into ferroptosis research establishes a foundation for exploring analogous roles of additional MEF2 family members. Targeting MEF2D or ACSL3 may represent a unique approach to counteract medication resistance, either by the application of small molecule inhibitors to disrupt MEF2D’s interaction with the ACSL3 promoter or by inhibiting ACSL3 activity using MUFA synthesis inhibitors (Henley and Koehler, 2021; Huang et al., 2025; Pisanu et al., 2018). Nonetheless, the potential toxicity to normal tissues, such as the heart and muscles, requires meticulous assessment. ACSL3 upregulation and ACSL4 downregulation correspond with ACSL4’s established pro-ferroptotic activity; however, confirmation is needed to determine if they exert functional antagonism via substrate competition (e.g., PUFA and MUFA). MEF2D has been previously identified as a promoter of HCC advancement through cell cycle regulation, and this study enhances its significance in metabolic control, indicating it may function as a “master regulator” that integrates many oncogenic signals. Future research should explore how this metabolic axis integrates with established resistance mechanisms, such as the hyperactivation of survival signaling pathways like PI3K/AKT or MAPK. For instance, MEF2D could act as a crucial downstream effector, translating oncogenic signals from these pathways into a specific metabolic rewiring program that confers resistance. This perspective positions the MEF2D-ACSL3 axis not merely as a standalone pathway but as a critical metabolic node within a larger, interconnected resistance network, thereby offering a novel therapeutic angle distinct from conventional kinase inhibitors. Notwithstanding advancements, obstacles in translational medicine, such as precision targeted delivery technologies, and the influence of the metabolic milieu on ACSL3 function persist as future problems. For example, the potential of exogenous MUFA to mitigate ferroptosis without affecting ACSL3, as well as the dynamic regulation of drug resistance by lipid supply in the tumour microenvironment, necessitate additional investigation. This study offers a theoretical foundation for the development of combination medicines; however, it requires confirmation in models that more closely resemble actual conditions to facilitate the move from mechanistic research to clinical application.

## Conclusion

This study elucidates the pivotal function of the MEF2D-ACSL3 axis in sorafenib resistance in HCC using multi-dimensional tests, establishing a theoretical basis for the development of combination therapies. Future investigations should meticulously examine the regulatory network of this axis and substantiate its targeting potential in clinically pertinent models to connect mechanistic research with therapeutic application.
